# Effects of ketogenic diet on the classification and functional composition of intestinal flora in children with mitochondrial epilepsy

**DOI:** 10.3389/fneur.2023.1237255

**Published:** 2023-07-31

**Authors:** Jing Wang, LIjuan Huang, Hua Li, Guohong Chen, Liming Yang, Dong Wang, Hong Han, Guo Zheng, Xu Wang, Jianmin Liang, Weijie He, Fang Fang, Jianxiang Liao, Dan Sun

**Affiliations:** ^1^Department of Pediatric Neurology, Wuhan Children’s Hospital, Tongji Medical College, Huazhong University of Science and Technology, Wuhan, Hubei, China; ^2^Department of Epilepsy Center, Guangdong 999 Brain Hospital, Guangzhou, China; ^3^Department of Neurology, Henan Provincial Children’s Hospital, Zhengzhou, China; ^4^Department of Neurology, Hunan Provincial Children’s Hospital, Changsha, China; ^5^Department of Neurology, Xi’an Children’s Hospital, Xi’an, China; ^6^Department of Neurology, Children’s Hospital of Shanxi, Taiyuan, China; ^7^Department of Neurology, Nanjing Children’s Hospital, Nanjing, China; ^8^Department of Neurology, Changchun Children’s Hospital, Changchun, China; ^9^Department of Neurology, The First Bethune Hospital of Jilin University, Changchun, China; ^10^Aegicare (Shenzhen) Technology Co., Ltd., Shenzhen, China; ^11^Department of Neurology, Beijing Children’s Hospital, Capital Medical University, Beijing, China; ^12^Department of Neurology, Shenzhen Children’s Hospital, Shenzhen, China

**Keywords:** mitochondrial epilepsy, ketogenic diet, gut microbiota, microbiota-gut-brain axis, *Bacteroides fragilis*

## Abstract

The ketogenic diet (KD) has shown excellent performance in the treatment of refractory epilepsy, but how it works is not yet fully understood. Gut microbiota is associated with various neurological disorders through the brain-gut axis. Different dietary patterns have different effects on the composition and function of gut microbiota. Here, by analyzing fecal samples from some patients with mitochondrial epilepsy before and after KD treatment through 16SrRNA sequencing, we found that KD intervention reduced the abundance of *Firmicutes* in the patient’s gut, while the abundance of *Bacteroidota* increased in the KD group. LefSe analysis showed that *Actinobacteriota*, *Phascolarctobacterium* had significant advantages in the control group, while Bacteroides increased significantly after KD intervention, especially *Bacteroides fragilis*. Functional analysis showed that there were significant differences in 12 pathways in level 3. These changes suggest that KD can change the composition and diversity of the gut microbiota in patients and affect their function. Changes in specific bacterial groups in the gut may serve as biomarkers for the therapeutic effects of KD on epilepsy.

## Introduction

The ketogenic diet is a low-carbohydrate, high-fat diet that induces the use of ketone bodies as the primary energy substrate by modulating intermediary metabolism. Developed in the 1920s (1), it has been widely used in the treatment of intractable epilepsy and has been shown to be safe and effective in this context (2–4). It is commonly believed that the KD plays an important role in mitochondrial biogenesis and function improvement (5), our previous studies have demonstrated that KD plays a significant role in the treatment of mitochondrial epilepsy (6) as well as in reducing oxidative stress (7–9). Increasing evidence suggests that the KD may also be involved in regulating brain excitability and epilepsy through pathways such as interrupting glutamate synaptic transmission (10), reducing glycolysis, and activating ATP-sensitive potassium channels (11). It is worth mentioning that due to its role in neuronal metabolism regulation, the KD has also been widely used in other neurological disorders such as Alzheimer’s disease and autism spectrum disorders (12). However, it is still unclear how KD exactly exerts beneficial effects on brain activity and behavior. The classical KD relies on nearly complete elimination of dietary carbohydrates to induce ketosis, allowing long-chain fatty acids (LCFAs) in the liver to produce ketone bodies. Currently, alternative ketogenic supplements, such as medium-chain triglycerides (MCTs), can rapidly induce ketone production, leading to extensive neuroprotective effects (13).

As an intermediary between diet and host, the composition and abundance of gut microbiota are regulated by various factors, such as internal factors like genetics and age, and external factors like region and diet (14). We know that the composition of gut microbiota can change rapidly and persistently with dietary changes (15, 16). In recent years, more and more researchers have paid attention to the changes in gut microbiota in normal and diseased populations, and studies have shown that they play a regulatory role in the progression of various diseases in the human body. For example, in obese populations, there are significant changes in the phyla Bacteroidetes and Firmicutes (17, 18); in non-alcoholic fatty liver disease and type 2 diabetes, the gut microbiota also plays an important mediating role (19). The changes in gut microbiota induced by KD may affect metabolic and neural pathways related to epilepsy (16), and there is also evidence that the use of antibiotics increases the risk of epileptic seizures (20). This suggests that gut microbiota may play a key role in alleviating symptoms of epilepsy in ways that we are not yet clear about. Xie et al. (21) found significant differences in the gut microbiota composition between 14 epileptic children and 30 healthy individuals, and observed a decrease in the abundance of dominant pathogens (such as *Escherichia coli*, *Salmonella*, and *Vibrio*) and a significant increase in beneficial bacteria (such as *Bacteroides*) in the gut of epileptic children after KD treatment. Zhang et al. (22) analyzed the gut microbiota composition of 20 refractory epilepsy patients before and after KD treatment, and found that the α-diversity of gut microbiota was lower after KD treatment, with a significant decrease in the abundance of *Firmicutes* and an increase in the abundance of *Bacteroidetes*. Lindefeldt et al. (23) studied the gut microbiota of 12 drug-resistant epilepsy children and found that after 3 months of KD treatment, there was no significant change in the α-diversity of the gut microbiota, but the abundance of *Bifidobacterium* was significantly reduced, and the abundance of *Escherichia coli* increased. Based on these studies, although KD treatment was administered to all epilepsy patients, there were differences in the source of dietary fat and the timing of intervention, as well as differences in patient age, race, and epilepsy etiology, which led to variations in gut microbiota composition before and after KD treatment. Therefore, it is difficult to compare the results and establish a consensus based on these studies.

Here, we aimed to explore the relationship and potential altered pathways between ketosis, gut microbiota, and mitochondrial epilepsy. We performed 16S rRNA sequencing on samples obtained after following a KD, analyzed changes in microbial abundance and diversity in various populations, and conducted enrichment analysis of the potential functional pathways they may be involved in to reveal their relationship with the alleviation of epilepsy symptoms.

## Materials and methods

### Study cohort

Between January 2019 and December 2020, a total of 15 patients with mitochondrial diseases accompanied by epilepsy, ranging from newborns to 16 years old (Supplementary Table 1). Mitochondrial diseases were confirmed through genetic diagnosis (identifying pathogenic gene mutations in mtDNA or nDNA) and abnormal biomarkers (lactate and ketone bodies). The epilepsy condition was assessed using the International League Against Epilepsy (ILAE) classification system (24). Patients meeting the following criteria were excluded: those who had previously received KD treatment, other genetic metabolic disorders, immunodeficiency, severe gastrointestinal, cardiovascular, respiratory, hepatic, or urogenital diseases.

### Experimental design

All eligible participants were randomly assigned to two groups (control group: regular diet + AED, study group: KD + AED) for treatment. During the enrollment period for starting KD, fecal samples were collected from the control group patients. Subsequently, KD was initiated at a 2:1 ratio of fat to non-fat components, with no fluid restriction and no fasting. The nutritionist calculated the daily energy requirements for each participant based on their age, height, and weight. Blood ketones and glucose levels were monitored every 6 h for the first 4 days. All side effects such as high ketone levels and low blood sugar were taken seriously and promptly addressed. Starting from the fifth day, energy intake was adjusted to meet daily requirements. During the baseline period, the nutritionist recorded the participants’ conditions using an observation chart. The first month of KD, known as the efficacy observation period or titration period, was a critical time for adjustments. The doctor or nutritionist made adjustments to the KD ratio, daily energy intake, meal times, meal frequency, and calorie intake based on the patient’s condition. At the end of the 12th week, the second fecal sample was collected. The control group followed a regular diet plus standard AED for the first 4 weeks. Afterward, they underwent 12 weeks of KD treatment alongside the study group. Parents could decide to withdraw from the study at any time due to poor efficacy, poor tolerance, or other reasons. The doctor also had the authority to terminate KD if the participant experienced side effects or disease exacerbation.

To compare the gut microbiota composition between the KD group and the control group, 16S rRNA analysis was conducted. Further research was carried out using methods such as diversity analysis, differential analysis, and functional prediction to explore the differences in gut microbiota composition and functional pathways between the two groups.

### Sample collection and storage

Fecal samples were obtained by parents using sterile cotton swabs and collection boxes. The first sample was collected before the initiation of KD. After 3 months of KD, a second sample was taken, which was then stored in a refrigerator for several hours until it was transported at low temperatures to the hospital. All samples were stored at −80°C.

### Sample processing and sequencing

After sample collection, according to the instructions of the E.Z.N.A.® soil DNA kit (Omega Bio-tek, Norcross, GA, United States), microbial community total DNA extraction was performed. The 16S rRNA gene V3-V4 variable region was amplified by PCR using the primers 338F (5′-ACTCCTACGGGAGGCAGCAG-3′) and 806R (5′-GGACTACHVGGGTWTCTAAT-3′). After mixing the PCR products, the mixture was purified using a 2% agarose gel and the AxyPrep DNA Gel Extraction Kit (Axygen Biosciences, Union City, CA, United States). Library construction was performed using the NEXTFLEX Rapid DNA-Seq Kit, and sequencing was carried out using the Illumina NovaSeq PE250 platform.

### Bioinformatics analysis

First, the software Trimmomatic v0.33 was used to filter the raw reads obtained from sequencing. Then, the software cutadapt 1.9.1 was used to identify and remove primer sequences, resulting in clean reads without primer sequences. The dada2 (25) method from QIIME2 2020.6 (26) was used for denoising, paired-end sequence merging, and removal of chimeric sequences, obtaining the final set of valid data (non-chimeric reads).

QIIME was also used for calculating the diversity of 16S rRNA gene sequencing analysis. Diversity indices (such as Shannon and Chao1) were used to evaluate the α-diversity of the samples. β-diversity was calculated based on the weighted Bray-Curtis distance matrix and visualized in principal coordinate analysis (PCoA). Linear discriminant analysis (LDA) effect size (LEfSe) was used to identify statistically significant differences in the relative abundance of taxa. Only LDA values >3 were considered significantly enriched. Functional prediction was performed using Tax4Fun, analyzing the composition and differences of metabolic pathways based on the KEGG database.

### Statistical analysis

Data analysis was performed using SPSS statistical software (SPSS, Chicago, IL, United States). Student’s *t*-test was used for comparing means between two groups, and the chi-square test was used for analyzing categorical variables. Measurement data are presented as mean ± standard deviation. Spearman correlation analysis was used to evaluate the relationship between gut microbiota and epilepsy (*p* < 0.05 considered significant).

## Results

### The demographic and clinical characteristics

Fifteen patients with mitochondrial epilepsy from 11 clinical centers participated in this study. They were randomly divided into two groups: eight in the KD group and seven in the control group. There were no significant differences in gender, age, duration of illness, medication, and number of seizures between the two groups (*p* > 0.05). We collected fecal samples from the patients at two time points: before starting KD and 3 months after starting KD. We obtained seven control group samples and eight KD group samples in total (Table 1).

**Table 1:** Demographic characteristics of the study population between the KD and control groups.

	KD group (*n* = 8)	Control group (*n* = 7)	*p* value
Age (year)	8.4 ± 4.8	4.7 ± 4.5	0.174
Sex			
Male	7 (87.5%)	6 (85.7%)	0.919
Female	1 (12.5%)	1 (14.3%)	
Age of onset (year)	6.9 ± 4.3	3.3 ± 3.8	0.135
Course of disease (year)	1.5 ± 1.0	1.3 ± 1.0	0.797
Seizure type			
Focal onset	4 (50.0%)	5 (71.4%)	0.398
Generalized onset	4 (50.0%)	2 (28.6%)	
Frequency (seizures per month)			
0–4	5 (62.5%)	2 (28.6%)	0.189
5–12	3 (37.5%)	5 (71.4%)	
Number of AEDs used (at the time of enrollment)			
0	3 (37.5%)	3 (42.8%)	0.966
1	1 (12.5%)	1 (14.3%)	
2	2 (25.0%)	2 (28.6%)	
4	2 (25.0%)	1 (14.3%)	

### Gut microbiota diversity

**Figure 1:**
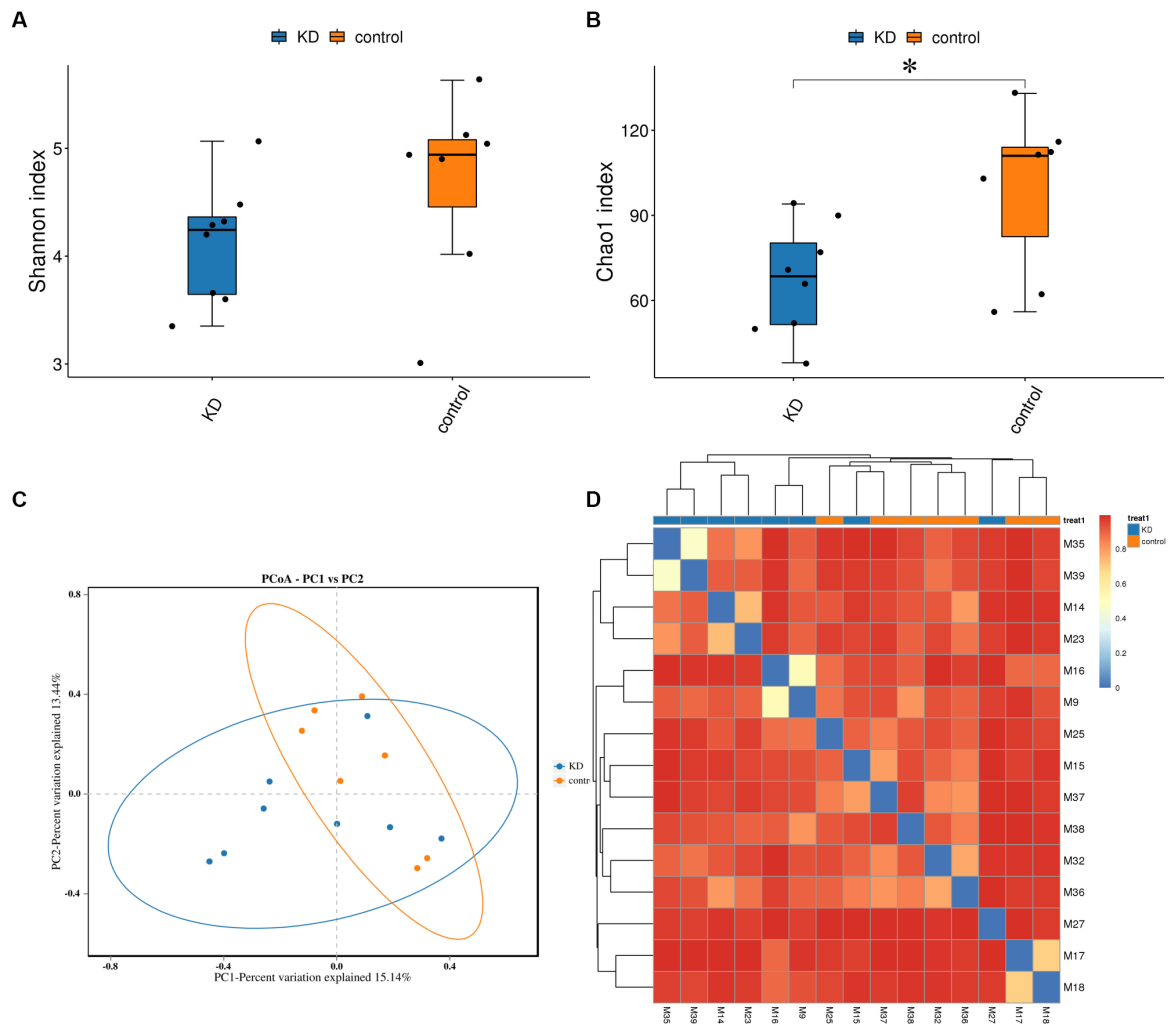
Diversity analysis of gut microbiota and microbial abundance in the KD group and the control group. **(A,B)** Alpha diversity (Shannon index) indicates no significant difference in gut microbiota diversity between the KD group and the control group (*p* > 0.05, calculated using Wilcoxon rank-sum test). Alpha diversity (Chao1 index) indicates significant difference in gut microbiota diversity between the KD group and the control group (*p* < 0.05). **(C)** Beta diversity PCoA analysis based on weighted Bray-Curtis distance indicates differences in gut microbiota composition between the KD group and the control group (blue dots represent the KD group, orange dots represent the control group). **(D)** Sample clustering heatmap based on weighted Bray-Curtis distance shows significant differences between the KD group and the control group. *p < 0.05.

**Figure 2:**
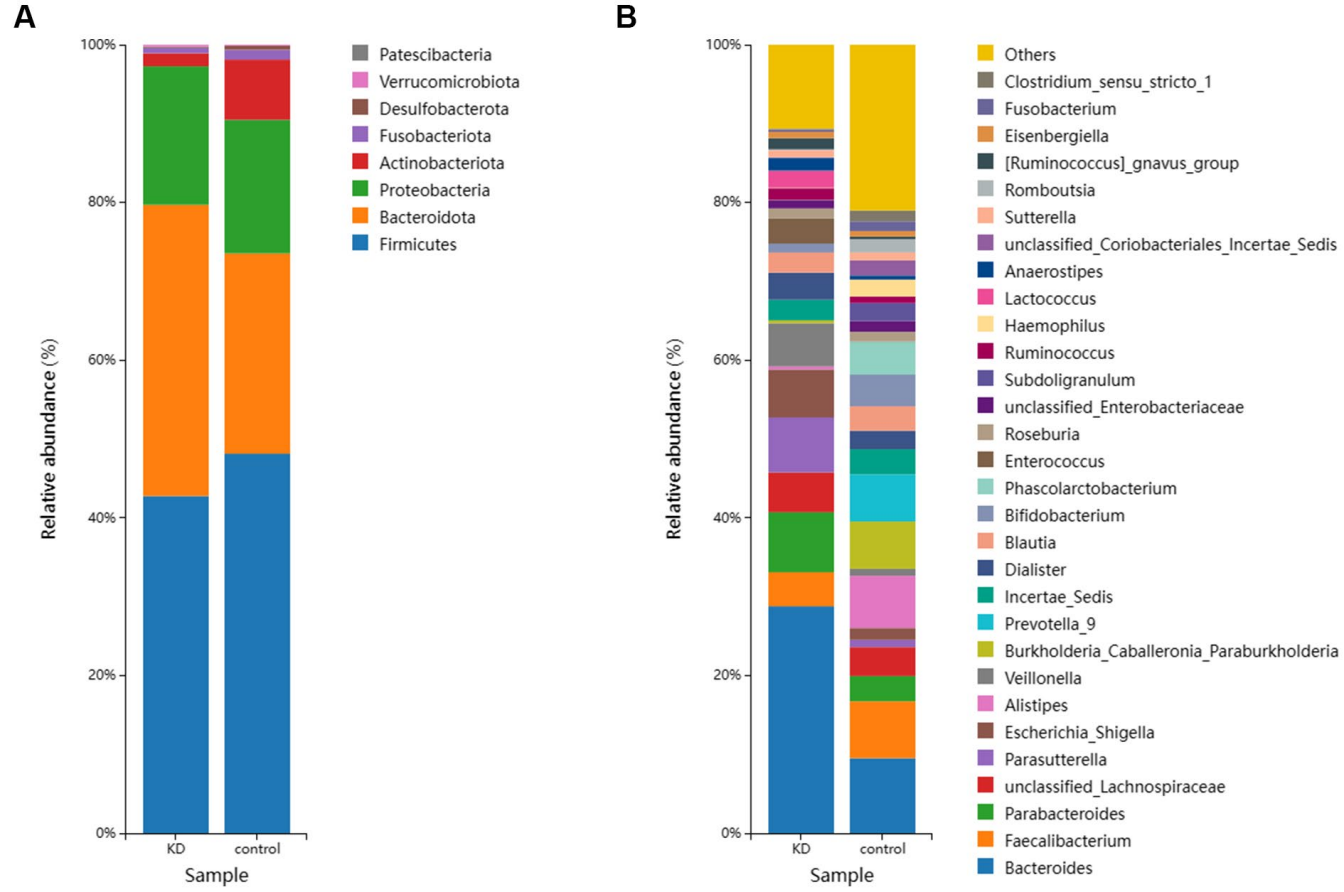
**(A)** The relative abundance of the top 10 microorganisms at the Phylum level in the KD group and the control group. **(B)** The relative abundance of the top 30 microorganisms at the genus level in the KD group and the control group.

To investigate the effect of ketogenic intervention on gut microbiota in patients, alpha diversity and beta diversity were used to reflect the diversity of the microbial community within samples and the differences between samples, respectively. We observed that Shannon diversity was slightly higher in the control group than in the ketogenic intervention group, but this difference was not significant (*p* > 0.05), while Chao1 diversity showed that microbial diversity was significantly higher in the control group than in the KD group (*p* < 0.05; Figures 1A,B). Beta diversity PCoA analysis based on weighted Bray-Curtis distance showed differences in microbial composition between the KD group and the control group (Figure 1C). The cluster heatmap also showed clear distinctions between the two groups of samples (Figure 1D). At the phylum level, *Firmicutes* was the largest phylum in both the KD group and the control group, but its abundance was relatively lower in the KD group (42.76% in the KD group vs. 48.13% in the control group). The second-largest phylum, *Bacteroidota*, showed a significant increase in abundance in the KD group (36.93% in the KD group vs. 25.41% in the control group). Some low-abundance microbes showed an increasing trend in the control group, including *Actinobacteriota* (1.66% in the KD group vs. 7.64% in the control group), *Fusobacteriota* (0.68% in the KD group vs. 1.65% in the control group), and *Desulfobacterota* (0.15% in the KD group vs. 0.50% in the control group; Figure 2A). At the genus level, compared to the control group, *Bacteroides* showed a significant increase in the KD group (28.78% in the KD group vs. 9.51% in the control group; Figure 2B).

**Figure 3:**
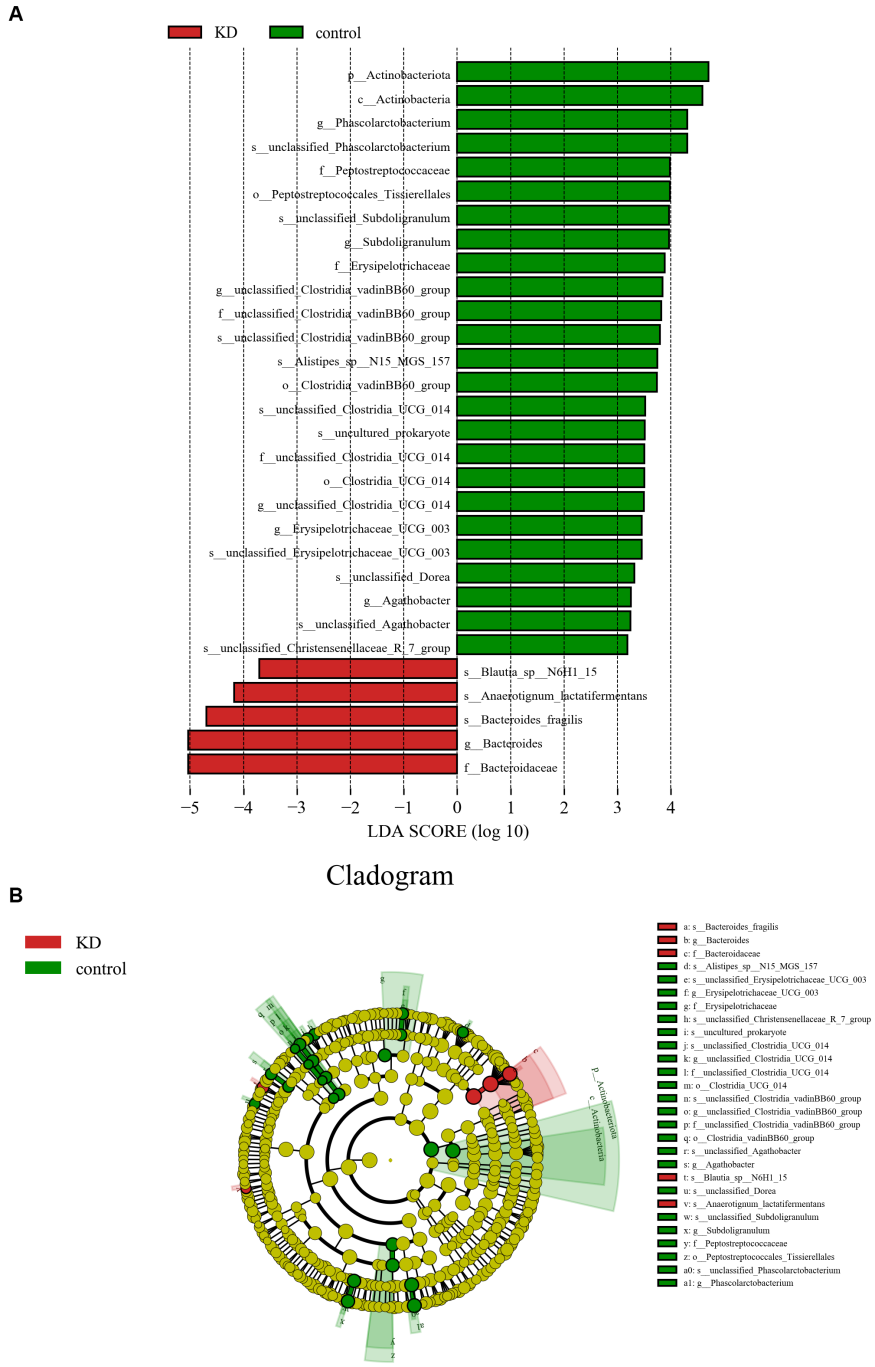
Analysis of differences in gut microbiota between KD group and control group. **(A)** LEfSe analysis: the length of the bars represents the logarithm of linear discriminant analysis (LDA). Green bars represent enriched microbiota in the control group; red bars represent enriched microbiota in the KD group (only species with LDA ≥ 3.0 are shown). **(B)** Cladogram of the phylogenetic distribution of microbes, microbes with an LDA value ≥3.0 in KD and control groups are marked with red and green.

We used the Linear Discriminant Analysis (LDA) Effect Size (LEfSe) method proposed by Segata et al. (27) to confirm the differences between the KD group and the control group. In the control group, the abundance of *Actinobacteriota* increased significantly at the phylum level, while the abundance of *Firmicutes* and *Bacteroidota* changed without significant differences (*p* > 0.05). The abundance of *Phascolarctobacterium*, *Subdoligranulum*, *Agathobacter*, and *Erysipelotrichaceae_UCG_003* was relatively high at the genus level. In the KD group, the abundance of *Bacteroides* increased significantly at the genus level, mainly due to the significant increase of *Bacteroides fragilis* at the species level. We also observed an increase in *Blautia.s__Blautia_sp__N6H1_15* at the genus level and *Anaerotignum_lactatifermentans* at the species level in the KD group (Figures 3A,B).

### Changes in gut microbiota function

Tax4Fun was used for functional prediction. In the comparison between the KD group and the control group, we found some obvious differences in three levels of KEGG pathways. Specifically, at each level, we found that some metabolic pathways, such as Environmental Information Processing and Cellular Processes, were slightly downregulated in the KD group, but not significantly. However, in level 2, some pathways showed significant differences between the two groups, with Infectious diseases: Bacterial and Signal transduction significantly elevated in the KD group. Particularly in level 3, a total of 320 pathways showed differences, mainly enriched in metabolic pathways such as Biosynthesis of secondary metabolites, Biosynthesis of antibiotics, Microbial metabolism in diverse environments, Two-component system, ABC transporters, Biosynthesis of amino acids, Carbon metabolism, Quorum sensing, Amino sugar and nucleotide sugar metabolism, and Purine metabolism. There are 12 pathways with significant differences in the group. The KD group shows an increased enrichment in pathways such as Citrate cycle (TCA cycle), Pertussis, Phosphatidylinositol signaling system, Biofilm formation—*Escherichia coli*, Penicillin and cephalosporin biosynthesis, Lysosome, and Glycosphingolipid biosynthesis—lacto and neolacto series. On the other hand, a decreasing trend is observed in pathways such as Quorum sensing, Bacterial secretion system, Nicotinate and nicotinamide metabolism, Legionellosis, and Arginine biosynthesis. Meanwhile, differences between the two groups are also observed in some other highly enriched pathways, such as Phenylalanine metabolism and Phenylalanine, tyrosine, and tryptophan biosynthesis, in which the former shows a decrease and the latter shows an increase after KD (Supplementary Table 2).

## Discussion

Mitochondrial epilepsy is difficult to control with a single anti-epileptic drug due to its diverse forms and types of seizures. Usually, a combination of drugs is used, but in this case, the mitochondrial toxicity of the drugs should be taken into consideration, and drugs that interfere with the respiratory chain should be used with caution (28). Therefore, the introduction of a KD to alleviate the symptoms of epilepsy is an effective solution. Since the classic KD was adopted in 1920, variants such as the Atkins diet (MAD) and the medium-chain triglyceride diet (MCT) have been developed based on palatability and compliance (29). Although researchers have been exploring the molecular mechanisms of KD’s effects, there is still no clear conclusion. In recent years, many researchers have attempted to study the anti-epileptic effects of KD in animal models. KD changed the gut microbiota of epileptic mice, and this change was necessary for the mice to cope with seizures (30). This was confirmed in a patient with Crohn’s disease who was cured of epilepsy through fecal microbiota transplantation (FMT) and did not experience a recurrence of epilepsy in subsequent follow-ups (31). Meanwhile, in our previous research, 40.9% of participants achieved 50% seizure reduction after 3 month of diet intervention. The KD also showed high efficacy in participants with mitochondrial encephalopathy, lactic acidosis, and stroke-like episodes (MELAS) or pathogenic variants in mitochondrial DNA (mtDNA) (6).

We investigated the effects of a KD on the composition and abundance of gut microbiota in patients with mitochondrial epilepsy. All patients in the KD group underwent a 3-month intervention using a 2:1 KD. By analyzing the 16S rRNA sequencing of fecal samples, we found a significant increase in *Bacteroides* and its subspecies *Bacteroides fragilis* after the KD intervention, while *Firmicutes* showed a certain degree of decrease, which is consistent with the results of Alina Arulsamy et al. (32) (Figures 2A,B). Although there was no significant difference in alpha diversity between the two groups, the beta diversity and clustering heatmap indicated some differences between the two groups (Figures 1C,D). In the LEfSe results, we found a significant enrichment of *Bacteroides fragilis* in the KD group, which directly led to the increase in *Bacteroides* abundance (Figure 3A). Previous mouse experiments have shown that oral treatment with human commensal *Bacteroides fragilis* can correct intestinal permeability, change microbiota composition, and improve defects in ASD-related communication, stereotypy, anxiety-like, and sensorimotor behaviors in MIA offspring (33). The induced changes in the symbiotic microbiota and serum metabolites may be the reason for this result. *Bacteroides fragilis* has been shown to play a significant role in other neurological diseases, and its bacterial capsule polysaccharide Ag can prevent autoimmune encephalitis (34). A previous study showed that compared to healthy individuals, the abundance of *Bacteroides fragilis* was lower in the gut of drug-resistant epilepsy patients (35). Similarly, this result was observed in a prospective study, in which *Bacteroides fragilis* played a significant role in the adjuvant treatment of drug-resistant epilepsy, with a reduction in seizure frequency of more than 53% in about 61% of patients (36). This suggests that *Bacteroides fragilis* may have a potential protective effect on seizures and may play a role as a key bacterium in the KD’s efficacy in epilepsy treatment. In addition, *Actinobacteriota* was significantly enriched in the control group, and Kihyun Lee identified it as a biomarker for epilepsy patients at the phylum level (37). This result has also been confirmed in many other studies (38, 39).

In terms of functional enrichment in the microbiome, level 1 did not show significant differences, and only two pathways, Infectious diseases: Bacterial and Signal transduction, showed differential enrichment in level 2. In level 3, a total of 320 metabolic pathways showed varying degrees of changes, with 12 showing significant differences, some of which were significantly decreased in enrichment after KD. Of particular interest is the Purine metabolism pathway, which was severely affected—almost all enzymes showed varying degrees of changes (Supplementary Figure 1). In previous studies, purines have been found to play important roles not only as a crucial component of cellular metabolism, but also in signal transduction. Susan A. Masino’s research has shown that the energy metabolism changes induced by the KD increase levels of ATP and adenosine, both of which may be the main mediators of the neuroprotective effects of the KD (40). Adenosine has been reported to be a powerful molecule for reducing seizures and providing neuroprotection (41). Our study only indirectly shows this possible effect, and more evidence is needed to prove changes in key substances or receptors. In Susan A. Masino’s recent study, KD was found to reduce seizures in mice by increasing the activation of adenosine A1 receptors (A1Rs) (42). Several pathways, including Arginine biosynthesis, Cysteine and methionine metabolism, and Valine, leucine and isoleucine biosynthesis, were also affected to varying degrees in our KD-treated group, and it is not yet clear whether this is related to the amino acid metabolic disorders associated with epilepsy (43). As a pathway with a relatively high level of enrichment, ABC transporters were higher in the control group than in the KD group, but not significantly. In previous studies, drug-resistant epilepsy patients showed a significant increase in the abundance of ABC transporter proteins, which is consistent with our results (35).

In general, our study reveals the impact of the KD on the gut microbiota in mitochondrial epilepsy patients. These findings suggest its potential role in epilepsy relief. However, for this study, we collected a limited number of cases, and accumulating cases proved to be a challenging process. The limitation in sample size restricts the conclusions drawn from our research. One contributing factor is the difficulty in implementing the KD in clinical settings, considering patient compliance and adverse effects. It is necessary for us to further investigate the specific effects of microbiota changes at the metabolite level, as this could provide a more direct explanation for the interaction between the gut microbiota and the nervous system. Similarly, we are curious whether extending the duration of KD would have a more pronounced impact on the gut microbiota, but these results require further research and observation. We hope that subsequent studies can uncover the relationship between the gut microbiota and the nervous system, providing a theoretical basis for the treatment of mitochondrial epilepsy.

## Data availability statement

The original contributions presented in the study are included in the article/Supplementary material, further inquiries can be directed to the corresponding authors.

## Ethics statement

The studies involving human participants were reviewed and approved by the Ethics Review Committee of Wuhan Children’s Hospital and the ethics review number is 2021R048-F01. Written informed consent to participate in this study was provided by the participants’ legal guardian/next of kin.

## Author contributions

DS, FF, JLiao, and LH designed the study and revised the manuscript accordingly. JW, HL, GC, LY, DW, HH, GZ, XW, JLiang, and WH participated in the study and provided case information. JW, LH, and DS drafted the manuscript. All authors contributed to the article and approved the submitted version.

## Funding

This study was supported by the Fund of Futang Research Center of Pediatric Development (No. FTCSF-2018-05), the Fund of Shenzhen Zeneca Biotechnology Co., Ltd. (No. 20181208), China Association Against Epilepsy (CAAE) Research Fund Qitong Fund (No. CJ-B-2021-20) and the Foundation under the designation CJ-B-2021-20.

## Conflict of interest

WH was employed by Aegicare (Shenzhen) Technology Co., Ltd.

The remaining authors declare that the research was conducted in the absence of any commercial or financial relationships that could be construed as a potential conflict of interest.

## Publisher’s note

All claims expressed in this article are solely those of the authors and do not necessarily represent those of their affiliated organizations, or those of the publisher, the editors and the reviewers. Any product that may be evaluated in this article, or claim that may be made by its manufacturer, is not guaranteed or endorsed by the publisher.
