# Supplementary material for: Effects of ketogenic diet on the classification and functional composition of intestinal flora in children with mitochondrial epilepsy
Source: Front Neurol. 2023 Jul 31;14:1237255. doi: 10.3389/fneur.2023.1237255 (PMC10426284; doi:10.3389/fneur.2023.1237255)
Supplement: Supplementary file 1 [file Data_Sheet_1.docx]

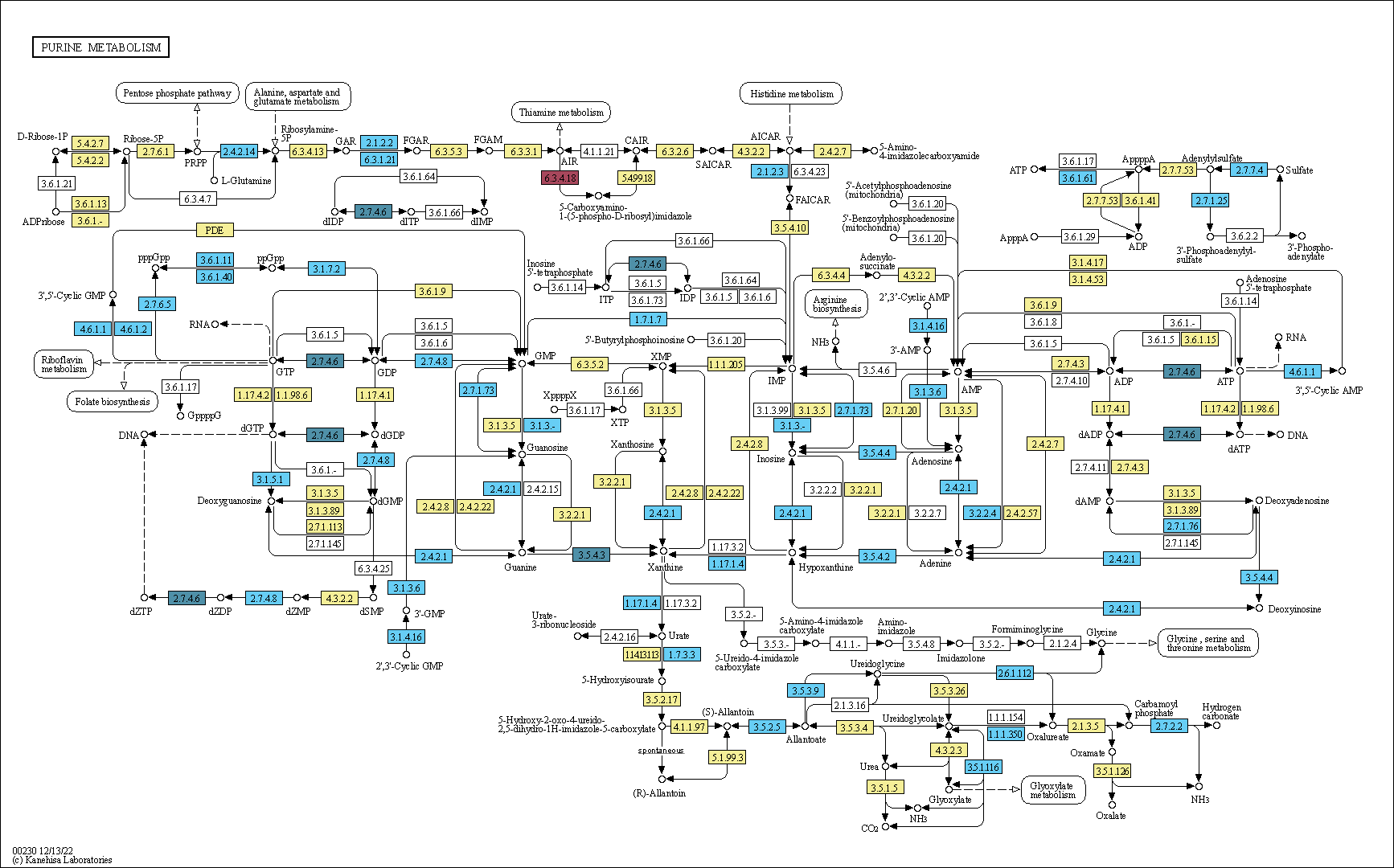


Supplementary figure 1. Enrichment differences in Purine metabolism, KD group>control group(*P*<0.05，dark blue), KD group>control group(*P*>0.05，blue), KD group<control group(*P*<0.05，red), KD group<control group(*P*<0.05，yellow).

| Supplementary table 1. Basic clinical information of patients in KD group and control group | | | | | | | |
| --- | --- | --- | --- | --- | --- | --- | --- |
| Group | Name | Gender | Age | Age of Onset Seizure | Type | Genetic Testing Results | |
| KD Group | p1 | male | 10Y7M | 10Y4M | Generalized onset | POLG | c.2558G>A c.2890C＞T |
|  | p2 | female | 6Y4M | 5Y1M | Generalized onset | MT-TL1 | m.3243A>G |
|  | p3 | male | 11Y8M | 10Y1M | Focal onset | MT-TL1 | m.3243A>G |
|  | p4 | male | 13Y3M | 11Y7M | Generalized onset | NA |  |
|  | p5 | male | 9Y11M | 7Y | Focal onset | SPG7 | c.1909G>A |
|  | p6 | male | 1Y5M | 3M | Focal onset | MT-ND5 | m.12770A>G |
|  | p7 | male | 5M | 3M | Generalized onset | PDHB | c.575G>T |
|  | p8 | male | 13Y8M | 10Y10M | Focal onset | MT-TL1 | m.3243A›G |
| Control Group | p9 | male | 4M | 40D | Focal onset | COQ4 | c.550T>C c.734T>C |
|  | p10 | male | 11Y10M | 9Y | Generalized onset | MT-TL1 | m.3243A>G |
|  | p11 | male | 5Y11M | 5Y | Generalized onset | MT-TL1 | m.3243A>G |
|  | p12 | male | 1Y10M | 3M | Focal onset | ATP6 | c.8993T>G |
|  | p13 | male | 10Y9M | 8Y4M | Focal onset | MT-TL1 | m.3243A›G |
|  | p14 | female | 1Y2M | 2M | Focal onset | QARS | C.1852G>A C.2068 C>T |
|  | p15 | male | 9M | 3M25D | Focal onset | FARS2 | c.424G>T c.998C>T |

| Supplementary table 2. Significant functional changes at Tax4Fun | | | |
| --- | --- | --- | --- |
|  | KD: mean(%) | control: mean(%) | *P*-values |
| **class2** |  |  |  |
| Infectious diseases: Bacterial | 0.626873643 | 0.511938828 | 0.035202963 |
| Signal transduction | 5.922560174 | 5.068045142 | 0.04706575 |
| **class3** |  |  |  |
| Citrate cycle (TCA cycle) | 0.619552073 | 0.515799038 | 0.006043665 |
| Quorum sensing | 1.956075786 | 2.588585388 | 0.00732704 |
| Bacterial secretion system | 0.476345478 | 0.554557982 | 0.017529193 |
| Nicotinate and nicotinamide metabolism | 0.353831864 | 0.446083691 | 0.023706248 |
| Pertussis | 0.281384831 | 0.16055201 | 0.024195354 |
| Phosphatidylinositol signaling system | 0.05615538 | 0.047295496 | 0.02480306 |
| Legionellosis | 0.087043969 | 0.108370288 | 0.025624924 |
| Biofilm formation - Escherichia coli | 0.807501751 | 0.629171767 | 0.036871409 |
| Arginine biosynthesis | 0.376496046 | 0.415973086 | 0.040373202 |
| Penicillin and cephalosporin biosynthesis | 0.026512649 | 0.015884254 | 0.040655239 |
| Lysosome | 0.269531659 | 0.123985129 | 0.042095866 |
| Glycosphingolipid biosynthesis - lacto and neolacto series | 0.003155874 | 0.000086 | 0.04989682 |
